# How Does Variation in the Body Composition of Both Stimuli and Participant Modulate Self-Estimates of Men’s Body Size?

**DOI:** 10.3389/fpsyt.2019.00720

**Published:** 2019-10-09

**Authors:** Vicki Groves, Piers Cornelissen, Kristofor McCarty, Sophie Mohamed, Nadia Maalin, Martin James Tovée, Katri Cornelissen

**Affiliations:** ^1^Department of Psychology, Northumbria University, Newcastle upon Tyne, United Kingdom; ^2^School of Psychology, University of Lincoln, Lincoln, United Kingdom

**Keywords:** male body image, body size estimation, body composition, muscularity, adiposity

## Abstract

When measured in units of body mass index (BMI), how much variation in men’s self-estimates of body size is caused by i) variation in participants’ body composition and ii) variation in the apparent muscle mass and muscle tone of the stimuli being judged? To address this, we generated nine sets of male CGI bodies representing low, mid, and high muscle mass rendered at low, mid, and high muscle tone, from 18.75 to 40 BMI_hse_ units. BMI_hse_ units in this study are estimates of BMI derived from calibration equations predicting BMI from waist and hip circumference, age, sex, height, and ethnicity in the Health Survey for England databases. Forty-five healthy adult men estimated their body size using a yes-no paradigm for each combination of muscle mass/tone. We also measured participants’ body composition with Harpenden callipers and their body concerns with psychometric questionnaires. We show that stimulus variation in apparent muscle mass/tone can introduce differences up to ∼2.5 BMI_hse_ units in men’s self-estimates of body size. Moreover, men with the same actual BMI, but different body composition, showed up to ∼5-7 BMI_hse_ unit differences in self-estimates of body size. In the face of such large errors, we advocate that such judgments in men should be made instead by simultaneously manipulating both the adiposity *and* the muscle mass of stimuli which are appropriately calibrated for body composition, so that the participant can match the body size and shape they believe themselves to have to the stimulus they see.

## Introduction

Several meta-analyses support the view that body image comprises i) a perceptual component which represents the accuracy with which a person can judge the physical dimensions of their own body and ii) an attitudinal component which captures the feelings that a person has about their body size and shape (1–4). Most people with anorexia nervosa (AN) experience body image distortion (DSM-5, 2013). Perceptually, they over-estimate their body size () and attitudinally, they have negative feelings towards their body. Persistent over-estimation and disparagement of own body size predicts the long-term outcome in treatment of AN (7, 9, 10), and its continuation post-treatment is a key predictor of relapse, which can be as high as 31% (11).

In men, not only is the incidence of anorexia nervosa rising (12, 13), but an increase in a drive for muscularity can lead both to the development of anorexia nervosa (14) and the onset of muscle dysmorphia, originally termed reverse anorexia (15). The presentation of male body image disorders shows a split between men who strive for thinness and those seeking to increase muscularity (16–18). Research also demonstrates that behaviors of men and women diagnosed with an eating disorder are more comparable than previously realized (19). This emphasizes the need for an accurate method for men to estimate their own body size. However, this measurement is problematic.

Men’s bodies vary in body composition (i.e., the relative proportion of total fat versus skeletal muscle mass) considerably more than women’s do. Yet virtually all scales developed for self-estimation of body size, to date, focus only on shape change that depends on percentage body fat, often expressed in units of BMI. However, the BMI itself is limited as an accurate index of body size and shape because it does not explicitly distinguish between the two principal dimensions of body composition, namely adiposity and muscle mass (20, 21). Since muscles are heavier than fat, increasing muscle mass makes a body denser, i.e. weighing more at the same volume (22). Consequently, the body shapes of two individuals with the same BMI, but different body composition, one with high and the other with low muscle mass, will be different. Moreover, in terms of clinical risk, a clinician would classify a man who weighs 148 kg and who is 1.93 m tall as severely obese according to World Health Organisation (23) criteria, because he would have a BMI of 39.7. But, if this individual had a lean body mass of ∼120 kg, a body fat percentage of less than 20%, and is a professional athlete, it can be assumed that he actually constitutes a low risk for obesity related disease (24).

In recognition of this measurement problem, attempts have been made to construct body scales for men which comprise systematically increasing combinations of muscle mass and adiposity. For example, Cafri and Thompson (25) constructed a line-drawn set of images based on the “somatomorphic matrix” (15). Arguably, these images lack realism in their depiction of individual bodies. More importantly, they showed low reliability on testing and the authors recommended their use should be discontinued (25). More recently Talbot, Smith, Cass, and Griffiths (26) produced a set of CGI bodies based on this image set (i.e., the new somatomorphic matrix). Although the authors found that the scores on their new measure showed good concurrent and convergent validity as a measure of male body dissatisfaction, as well as good test-retest reliability, they do not seem to have duplicated the size and shapes of these bodies in a formal, quantitative way. Specifically, there is no precisely calibrated mapping between the shape of the men in the images, and their body composition. The implication is, therefore, that while well intentioned, these somatomorphic matrices cannot currently be used to estimate body shape and size in men reliably.

## The Current Study

Here, we set out to measure how much variation in men’s self-estimates of body size (when measured in BMI_hse_ units) is caused by i) variation in participants’ own body composition and ii) variation in the apparent muscle mass and muscle tone of the stimuli being judged. To anticipate, our intention was to demonstrate that the sources of error in these measurements, when expressed in BMI_hse_ units, are large. Indeed, that the errors are likely to be so large that we really should, as a research community, be seeking to solve the problem by developing stimuli that are correctly calibrated for both muscle mass and body fat for use in body-size estimation tasks. This paper *does not* represent that ideal solution. Rather it is intended as a call to action, based on the quantitative evidence we present.

To achieve this goal, we have had to use an indirect strategy, because, to our knowledge, no large-scale database exists that would allow images to be generated that are correctly calibrated for body composition. Therefore, in this study, we used 3D CGI models of men that were independently judged to have qualitatively low, middle, or high muscle mass based on visual judgments alone. For each of the three sets of models, we allowed adiposity to vary continuously from a very slim figure through to a mildly obese figure. Any given level of adiposity can be assigned a BMI_hse_ value by substituting the model’s waist and hip circumference into a calibration equation derived from ∼5,000 observations from the Health Survey for England datasets [see, e.g., Refs. (27, 28)]. The key point here is that across the three levels of muscle mass, qualitatively determined, we can ensure that the waist and hip values are the same at every BMI_hse_ value, and that these circumferences increase at exactly the same rate with increasing BMI_hse_. This means that we can ask whether a participant who has a measured BMI of 26, for example, will match a stimulus to their own body that has the same BMI_hse_ across the three levels of qualitatively defined muscle mass, or different BMI_hse_ values. The possible outcomes can be explained by two alternative hypotheses.

The first hypothesis is based on behavioral and eye-movement studies of women making self-estimates of body size, which suggest that women judge BMI by estimating the width of the body in the abdominal region (29–31). Therefore, if men use the same gaze strategy when making self-estimates of body size as women, they could solve the tasks in the current study by identifying the stimuli which they believe to be a good match to their own waist and hip widths, i.e., making a match based on abdominal torso width. If this were the case, we would expect to see individuals choosing stimuli with the same BMI_hse_ values across the three levels of muscle mass reflecting the body size they believe themselves to have. Plots of the regression of BMI_hse_ on participants’ actual BMI would therefore produce overlapping regression lines with, statistically, the same slopes and intercepts. Any difference, or error, between actual BMI and the body-size estimate, expressed in BMI_hse_, should be equivalent across the three muscle mass levels.

An alternative, and we believe more likely hypothesis, assumes that men may use cues other than, or in addition to, torso edge separation (indexed by waist and hip widths), with which to make their judgments. For example, Crossley, Cornelissen, and Tovée (32) showed that males attach importance not only to the abdominal region but also the chest and the arms. Qualitative research conducted by Ridgeway and Tylka (33) questioned males about their ideal body composition and identified that as well as the upper body, males also discussed their thighs and calves as often as their shoulders and back. More recent eye tracking studies have confirmed the relevance of the chest, shoulders, and abdominal regions as areas of interest, not only when participants were looking at their own bodies, but when looking at other men (34, 35). Therefore, if men are using features like these to match the body-size/shape they believe themselves to have, then they may need, for example, to pick low muscle mass stimuli with higher BMI_hse_ values compared to the matches they make with high muscle mass stimuli. In other words, they may need to inflate the qualitatively lower muscle mass images in order to make a convincing match to their beliefs about their own body shape and size more than they do for higher muscle mass images. In this situation, plots of the regression of BMI_hse_ on participants’ actual BMI would produce non-overlapping regression lines with different intercepts, and possibly different slopes. Were we to find such effects, this would confirm that the qualitative visual properties of a stimulus set with respect to muscle mass and tone have a strong influence on estimates of body size.

In short, calibrating our stimuli based on the HSE datasets gives a BMI index (i.e., BMI_hse_) that is agnostic about the differential effects of muscle mass. However, because waist and hip circumferences can be held constant across the three qualitatively defined muscle mass levels, and by making sure that each participant repeats the task at each of these three muscle mass levels, we can infer something useful about the impact of stimulus muscle mass on participants’ body-size judgments by comparing between measurements, and expressing these difference in BMI_hse_ units. As a final step, we used a modestly sized body composition database of 178 men to assign plausible, quantitative muscle mass values to our stimuli, and thereby back calculate a likely real BMI value (this time sensitive to body composition) for them. We then repeated our analysis of the experimental data to test whether we converged on the same pattern of results.

In summary, we set out to measure how much error in men’s self-estimates of body size (when measured in BMI_hse_ units) is caused by i) variation in participants’ own body composition and ii) variation in the apparent muscle mass and muscle tone of the stimuli being judged. Additionally, we used a battery of standard psychometric measures to index participants’ psychological state and to allow us to factor this into our analysis.

## Methods

The experimental procedures and methods for participant recruitment for this study were approved by the local ethics committees at Northumbria University and the University of Lincoln. All experiments were performed in accordance with relevant guidelines and regulations set out by these organizations, and all participants gave their informed consent to take part in this study.

### Participants

An opportunity sample of 53 male participants aged 18–58 (M = 24.87, SD = 9.02) was recruited from a sample of university staff and students and individuals from surrounding areas. Following participation, eight participants were excluded from our data set either because they did not complete all nine psychophysical tasks or it proved impossible to compute adequate psychometric functions from their data in at least one task. Measures retrieved from a final sample of 45 male participants aged 18–58 (M = 24.73 years, SD = 9.23) were used for data analyses, 39 of whom consented to body-site measurements with Harpenden callipers (see **Table 1** for all participant characteristics). Participants were advised that their actual BMI should fall within the range from 18 to 40 to correspond with the BMI_hse_ range of stimuli sets. Individuals with a current diagnosis of an eating or body dysmorphic disorder were excluded from taking part in the research. There was no financial reward for taking part in the study.

**Table 1:** Descriptive statistics for age, actual BMI, body composition and questionnaire responses (n = 45).

	*M*	*SD*	Range	
			Actual	Potential
Participant characteristics				
Age (years)	24.73	9.23	18.00 – 58.00	
Actual BMI (kg/m^2^)	25.32	4.50	18.00 – 39.70	
Body fat (%)	19.75	3.69	13.94 – 30.37	
Skeletal muscle (%)	19.03	5.43	9.00 – 29.00	
Psychometric task performance				
BPSS-M	87.02	21.06	25.00 – 130.00	25 – 150
STQ Body fat	13.34	4.30	5.00 – 21.00	5 – 25
STQ Muscular	15.57	5.05	5.00 – 25.00	5 – 25
STQ Family pressure	7.09	3.79	4.00 – 19.00	4 – 20
STQ Peer pressure	7.84	3.25	4.00 – 16.00	4 – 20
STQ Media pressure	11.20	4.97	4.00 – 19.00	4 – 20
DMS Attitudes	26.18	9.21	7.00 – 42.00	7 – 42
DMS Behaviors	16.23	9.08	7.00 – 40.00	7 – 42
DMS Total	43.73	16.98	15.00 – 84.00	15 – 90

STQ, Sociocultural Attitudes Towards Appearance Questionnaire 4 (SATAQ 4); DMS, Drive for Muscularity Scale; BPSS-M, Body Parts Satisfaction Scale for Men.

### Psychometric Measurements

To assess participants’ current attitudes towards their body shape and size, the following questionnaires were used:

- *The Body Parts Satisfaction Scale for Men* (BPSS-M) (36). The 25-item BPSS-M asks participants to rate their level of satisfaction with their upper body, their face, and their legs on a scale from 1 to 6 (1 = *extremely dissatisfied*, 6 = *extremely satisfied*). The list of items includes both muscularity and leanness criteria, as well as an indication of an individual’s overall body satisfaction. For the purposes of this study, we reverse scored all items so that higher scores index a greater dissatisfaction with body size and shape.
- *The Sociocultural Attitudes Towards Appearance Questionnaire* (SATAQ-4) (37). The 22-item SATAQ-4 evaluates the extent of internalization of appearance ideals and appearance related pressures. The SATAQ-4 measures five subscales of one’s appearance: two for Internalization, consisting of thin/low body fat and muscular/athletic dimensions, and three for Pressures consisting of family, peers, and media dimensions. Items are rated on a Likert scale ranging from 1 to 5 (1 = *definitely disagree*, 5 = *definitely agree*), with higher scores indicating greater internalization and acceptance of societal appearance ideals.
- *The Drive for Muscularity Scale* (DMS) (38). Participant drive for muscularity was measured using this 15-item scale, which indexes two subscales of one’s muscularity drive: muscularity-oriented attitudes (7-items) and muscularity-related behaviors (7-items). The scale also provides an overall drive for muscularity score. Participants rated the items on a scale ranging from 1 to 6 (1 = *Always*, 6 = *Never*), and all items were reverse-coded so that higher composite scores indicated greater drive for, attitudes towards, and engagement in behavior to increase muscularity. Reliability testing for responses to the psychometric questionnaires across the sample showed good internal reliability.

For BPSS-M, SATAQ Body fat, SATAQ Muscular, SATAQ Family pressure, SATAQ Peer pressure, SATAQ Media pressure, DMS Attitudes, DMS Behavior, and DMS Total, Cronbach’s alpha was. 96, .75, .88, .90, .82, .94, .91, .92, and .94, respectively.

### Anthropometric Measurements

To assess participant’s current body size and shape, we used the following measures:

- *BMI*. This was measured using the same stadiometer and calibrated scales throughout the testing period and was calculated as BMI = Weight (kg)/Height (m)^2^.
- *Body Composition*. We used the Harpenden skinfold caliper as recommended by the International Standards for Anthropometric Assessment guide (ISAK) (39). Skinfold measurements (millimeters) were taken from eight key body sites: biceps, triceps, subscapular, iliac crest, abdominal, suprailium, mid-thigh, and medial calf; along with circumference measurements (cm) of the upper arm, mid-thigh, and calf, using a SECA 201 measuring tape. Body fat percentage was calculated using the final equation for men as set out by Peterson, Czerwinski, and Siervogel (40): % BF_new_ = 20.94878 + (age x 0.1166) – (height x 0.11666) + (sum4 x 0.42696) – (sum4^2^ x 0.00159), where height is in centimeters and sum4 is the sum of the triceps, subscapular, iliac crest, and mid-thigh skinfold thickness. Muscle mass percentage was calculated using the final equation developed by Lee et al. (41): SM (kg) = Ht x (0.00744 x CAG^2^ + 0.00088 x CTG^2^ + 0.00441 x CCG^2^) + 2.4 x sex – 0.048 x age + race + 7.8. This equation employed participants’ height (Ht), race (Caucasian/Hispanic = 0, Asian = 1, African American = 1.1), sex (male = 1, female = 0), corrected arm (CAG), thigh (CTG), and calf (CCG) girth measurements.

### Stimulus Generation

We created CGI images from the Genesis 8 male base model in a 3D modelling environment (DAZ Studio v4.8). The models stood in front of a virtual camera in three quarter view [cf. (42)]. This modelling environment allows adiposity, muscle mass, and muscle tone to be manipulated individually along separate morph dimensions. Based on pilot data, we picked three levels each for visually apparent muscle mass (low, mid, and high) and muscle tone (low, mid, and high) that 10 raters agreed constituted qualitatively distinct differences for these attributes across three BMI categories (underweight/healthy/overweight; 23). For these inter-rater judgments, the overall Kappa statistic for nominal judgment was 0.98 (SE = 0.035, Z = 28.29, p < .0001), suggesting that the qualitative differences between the three muscle mass and muscle tone renderings were indeed clear and unambiguous to participants. We then systematically manipulated the adiposity of the male model at each of the 9 muscle mass and muscle tone combinations, to produce a set of stimuli that varied in BMI_hse_ from 18.75 to 40 in 0.25 BMI_hse_ steps. We calibrated models for BMI_hse_ using the equation below, which was derived from the waist and hip circumference measurements from 5,705 Caucasian men, over the age of 18, from the HSE datasets (43). The height of the model to be entered into the calibration equation was 1.78 m [cf. (44)]. This calibration equation explains 88% of the variance relating the actual BMI of the 5,705 Caucasian men to their waist and hip circumferences, as well as their age and height:

$${\text{BMI}}_{\text{hse}}=\beta_{1}{\text{x}}_{1}+\beta_{2}{\text{x}}_{2}+\beta_{3}{\text{x}}_{3}+\beta_{4}{\text{x}}_{4}+\varepsilon$$

where x_1_ = waist circumference (cm), x_2_ = hip circumference (cm), x_3_ = height (cm), x_4_ = chronological age (years), β_1_ = 0.24 95% CI(0.23 – 0.25), β_2_ = 0.20 95% CI(0.19 – 0.21), β_3_ = -0.15 95% CI(-0.16 – -0.14), β_4_ = -0.024 95% CI(-0.047 – -0.042).

**Figure 1:**
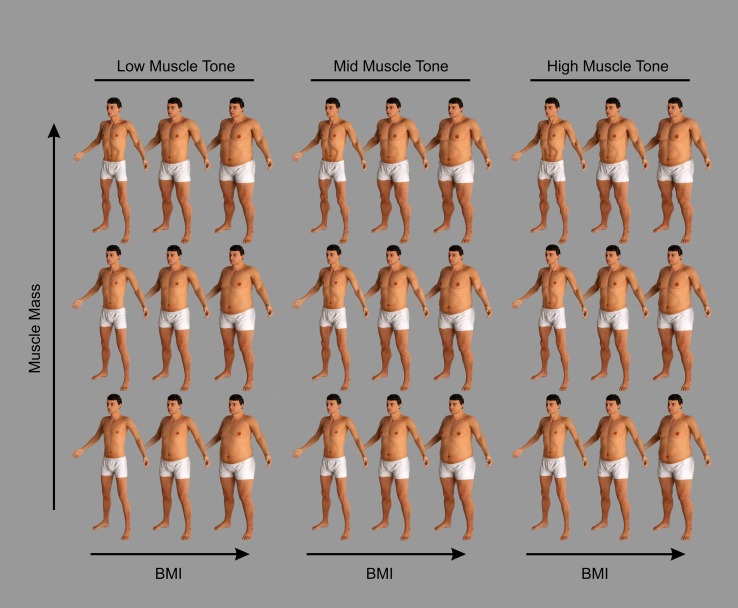
Examples of the CGI bodies used in this study to illustrate the changes of body shape and size of the male stimuli produced by changing their body composition. The images are grouped into three columns, from left to right: low, mid, and high muscle tone. They are further divided into three rows from bottom to top: low, mid, and high muscle mass. Ten raters agreed that these groupings constituted qualitatively distinct differences for these attributes across three BMI categories (underweight/healthy/overweight; 23). The overall Kappa statistic for nominal judgment was 0.98, SE = 0.035, Z = 28.29, p < .0001.

Individual stimulus images were ray-traced in Luxrender. The advantages of the stimuli sets are that the images i) are high definition and photorealistic, ii) maintain the identity of the male model across a wide BMI_hse_ range, and iii) demonstrate realistic changes in BMI_hse_ dependent body shape. Examples of the stimuli are shown in **Figure 1**. However, please note that, owing to the reduced contrast and resolution of this illustration, much image detail is lost compared to the original stimuli.

### Yes-No Psychophysical Task

**Figure 2:**
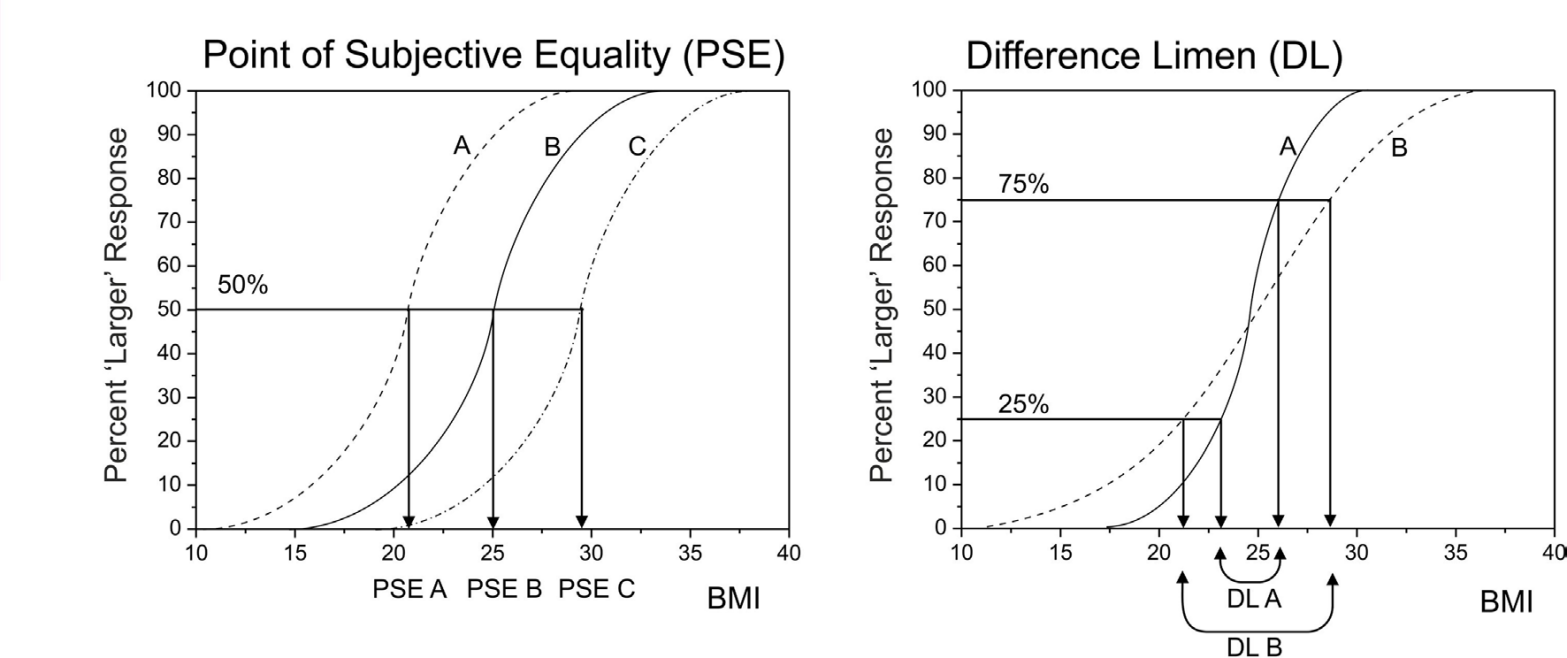
A graphical illustration of how the psychometric function for body size estimation can be used to separate out sensory sensitivity (indexed by the difference limen, DL) from perceptual bias (indexed by the point of subjective equality, PSE). On the left, participants A, B, and C might all have the same BMI of 25. However, participant A under-estimates and participant C over-estimates their body size. On the right, participant A is more sensitive to body size change than participant B, and therefore has a steeper psychometric function, with a smaller DL.

In this study we applied classical psychophysical methods [cf. (45)] to measure two components of the participants’ judgments of their own body size: i) the point of subjective equality (PSE) and ii) the difference limen (DL). The PSE is the participant’s subjective estimate of their body size, in this case measured in BMI_hse_ units. The DL is an estimate of how sensitive a participant is to changes in body size and equates to the smallest difference in body size that he can detect, again measured in BMI_hse_ units. To obtain these measurements, we used the method of constant stimuli in a yes-no forced choice paradigm. This allows a psychometric function to be estimated. Here, the psychometric function is a plot of the percentage of “this image is larger than me responses” as a function of the BMI_hse_ of the stimuli presented, and the curve tends to have a sigmoidal shape. The PSE is defined from the psychometric function as the BMI_hse_ at which participants would respond “larger than me” 50% of the time. The DL is the average of the differences in BMI_hse_ of the stimuli falling between the 25% and 50% and the 75% and 50% “larger than me” response points [see (46)]. This range captures the steepness of the psychometric curve. Participants who are very sensitive to small changes in body size will have a steeper psychometric function with a correspondingly small DL. **Figure 2** shows sketch plots to illustrate how the PSE and DL are derived from the psychometric function.

Participants carried out the yes-no task nine times, once for each combination of muscle mass/muscle tone. The order of presentation of muscle mass/muscle tone stimuli was randomized for each participant. For each yes-no task, participants were presented with a randomized sequence of images of the standard CGI male body model. Across the image set, BMI_hse_ varied continuously from 18.75 to 40.0. On each trial of the task, one image was presented and participants were required to decide whether the body depicted was larger than they were and to record this decision by button press (one button for “yes” and a second button for “no”). Stimuli were presented on a 19” flat panel LCD screen (1280w x 1024h pixel native resolution, 32-bit color depth) for as long as it took participants to make a decision. At the standard viewing distance of ∼60 cm, the image frame containing the male body subtended ∼26° vertically and ∼8° degrees horizontally. Each participant first judged seven images covering the whole BMI_hse_ range (from 18.75 to 40.0 in equal BMI_hse_ steps) presented in two separate blocks. Each stimulus image appeared 10 times in each block, and the order of presentation was randomized. Based on the responses from each block, the participants’ PSE (i.e., an estimate of the BMI_hse_ they believe themselves to be) was calculated automatically by fitting a cumulative normal distribution. These two values were then averaged to give an initial estimate of the participant’s PSE. On the basis of this initial estimate, the program presented a further set of 21 images (spread over a range of 5 BMI_hse_ units centered on the participant’s initial PSE, at a spacing of 0.25 units per image) for the participants to judge. Each image was presented 10 times in randomized order. This final set of judgments allowed us to plot the full psychometric function and use probit analysis off-line to calculate a definitive estimate of PSE as well as the DL (i.e., how sensitive participants are to changes in BMI_hse_).

### Timeline for Task Administration

Due to the nature of testing and the length of each task, participants were invited to take part over two testing sessions. During the first testing session, participants were invited to complete all psychometric questionnaires, before having their height and weight measured by the researcher in order to calculate actual BMI. Standardized verbal instructions were then given for the psychophysical tasks and participants were asked to complete the first four levels of the psychophysical task. Order exposure of task level was randomized. During the second session, which occurred within two weeks of the first, participants completed the remaining five levels of the psychophysical task. For participants who chose to participate, body composition measurements were also taken during the second session. Collectively, participation lasted approximately two hours.

### Analysis Pipeline

**Figure 3:**
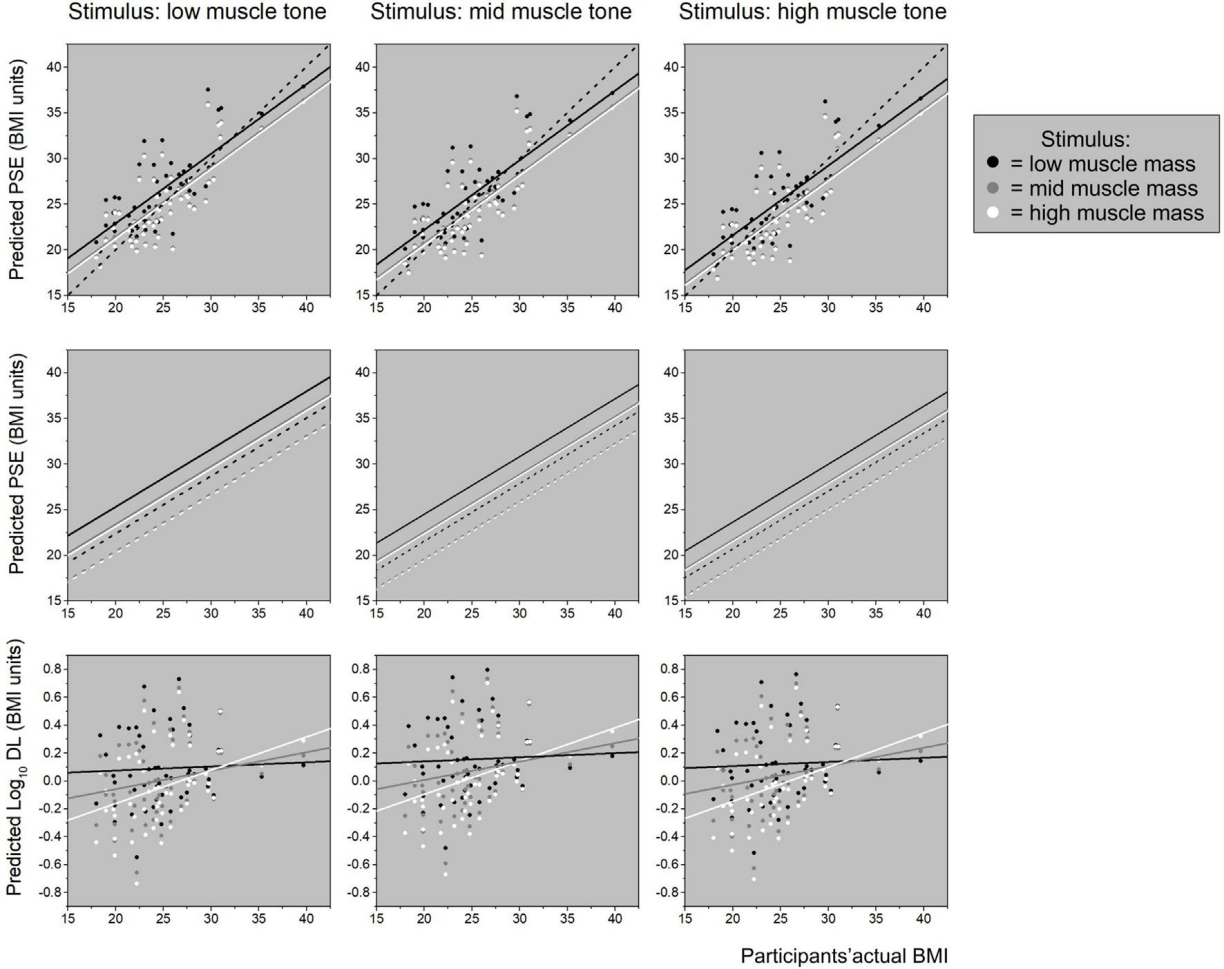
The *top row* shows three plots of body-size estimates (PSE) predicted from the linear mixed effect model (“Model 1 for PSE” shown in **Table 4**) plotted as a function of participants’ actual BMI. In each plot, low, mid, and high stimulus muscle mass is represented by black, gray, and white dots, respectively. The regression lines for each level of stimulus muscle mass follow the same color scheme. The plot on the left is for low stimulus muscle tone stimuli, the middle plot for mid stimulus muscle tone, and the plot on the right for high stimulus muscle tone. In each case, the black dashed line represents the line of equality, where body-size estimates (PSE) exactly match actual BMI. The graphs in the *middle row* show the same regressions of body-size (PSE) on actual BMI (from “Model 1 for PSE”, shown in **Table 4**) at the same three stimulus muscle mass levels within each plot, separately for the three stimulus muscle tone levels across the row, from low to high. However, now each regression line is split, and plotted separately at +1 SD (solid lines) and -1 SD (dashed lines) for Participant_Musc_Att, to illustrate the independent influence of participants’ psychometric performance on body size estimation. Specifically, increasingly positive attitudes and drive towards muscularity are associated with higher body size estimates. The *bottom row* shows three plots of participants’ sensitivity in the body size estimation task (i.e., DL) predicted from the linear mixed effect model (“Model 3 for PSE” shown in **Table 4**) plotted as a function of participants’ actual BMI. Each plot contains the predicted DL values and regression lines for low (black), mid (gray), and high (white) stimulus muscle mass as a function of actual BMI. Stimulus muscle tone changes from low, through mid, to high across the 3 plots from left to right. These graphs show that sensitivity to changing body-size systematically decreases as a function of increasing BMI, and that this effect is weakest for low muscle mass stimuli, intermediate for mid muscle mass stimuli, and strongest for high muscle mass stimuli.

**Figures 4:**
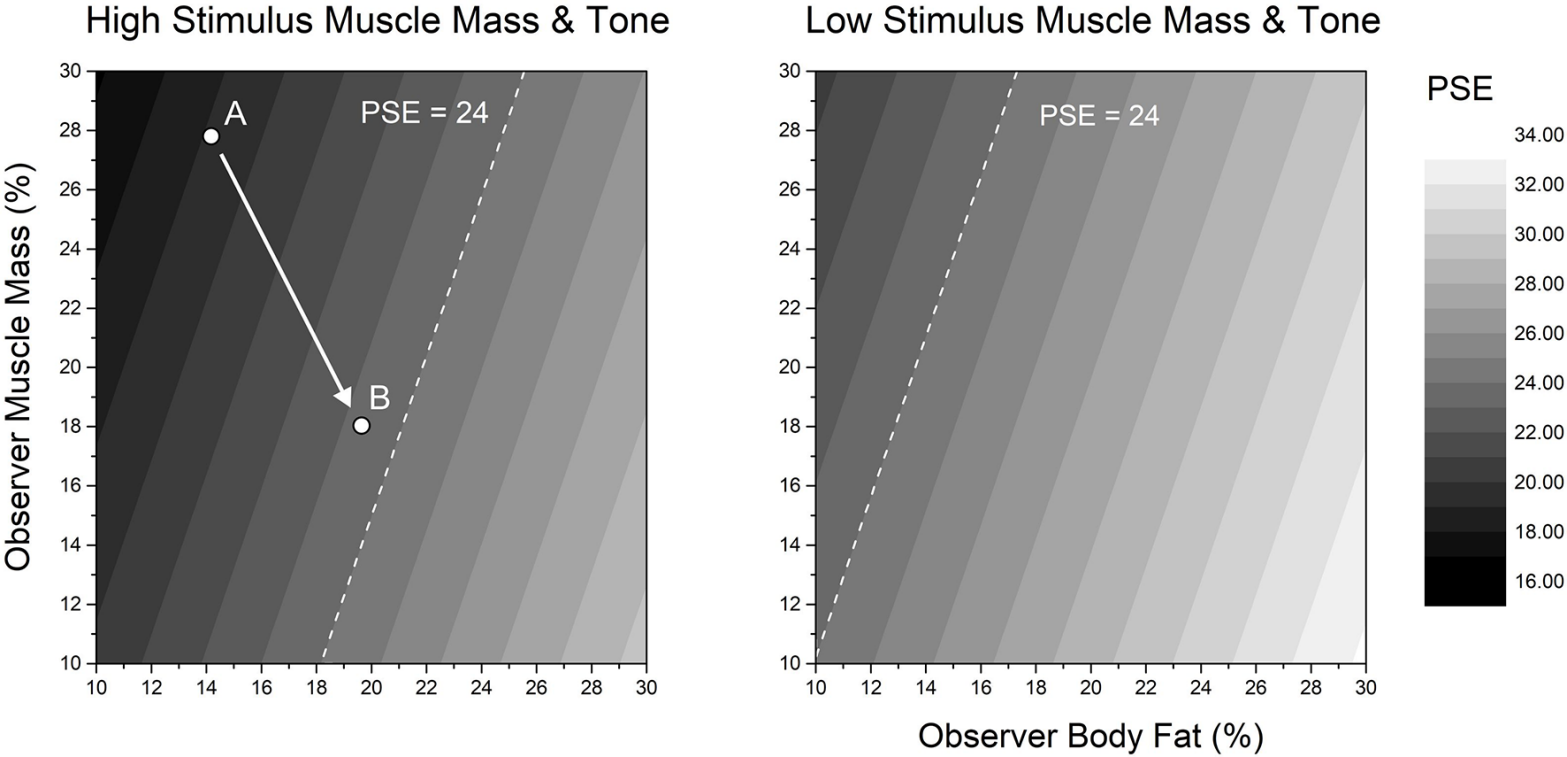
Two contour plots of body-size estimates (PSE) predicted from the linear mixed effect model (“Model 2 for PSE” shown in **Table 4**) plotted as a function of participants’ participant muscle mass (*y*-axis) and body fat (*x*-axis). Predicted body-size estimates (PSE) are represented in grey levels on the *z*-axis, from black (smaller body size) to white (larger body size). Responses from high stimulus muscle mass and high stimulus muscle tone are shown in the left panel. Responses from low stimulus muscle mass and low stimulus muscle tone are shown in the right panel. In both panels, as participants’ muscle mass increases, so the size that they believe themselves to be tends to decrease. Conversely, as participants’ body fat increases, so the body size that they believe themselves to have increases. Since estimated body-size changes in opposite directions for participant muscle mass and body fat, it is possible for differing body compositions to give rise to the same body size estimate. This is illustrated by the white dashed line in each plot, which corresponds to a predicted body size of 24 BMI_hse_. The converse of this situation is illustrated by points *A* and *B* in the left panel, which show the different muscle mass and body fat combinations from two participants in our dataset both of whom had an actual BMI ∼23.

**Figure 5:**
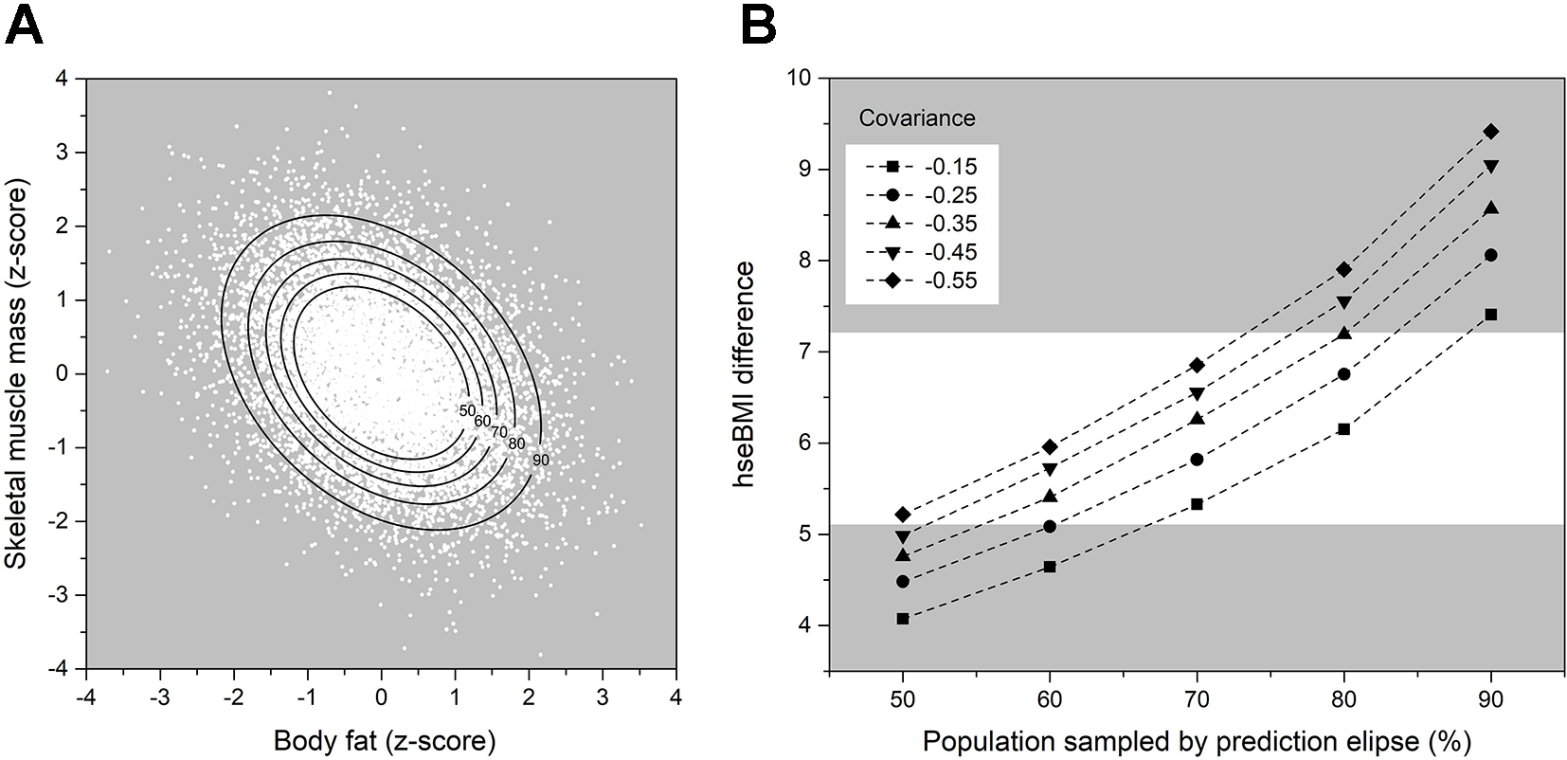
**(A)** Scatter plot of the 10,000 data point bivariate normal distribution for a covariance of 0.55 between percentage muscle mass and body fat (expressed as z-scores). The black lines represent prediction ellipses that capture, respectively, 50, 60, 70, 80, and 90% of the observations in the distribution. **(B)** Differences in estimated BMI (*y*-axis) between pairs of participants who would both have the same actual BMI, but differing body compositions. The range of these differing body compositions is determined by the particular combination of the covariance between body fat and skeletal muscle mass (to produce the bivariate distribution) and the prediction ellipse (which selects how many observations are chosen from the distribution). The white band highlights the most likely combinations of covariance and prediction ellipse parameters. See text for further details.

The main analyses of the experimental data included the following steps:

- Calculation and tabulation of univariate descriptive statistics for participants’ characteristics and their psychometric performance.
- Data reduction of the psychometric responses, using principal components analysis, to produce two latent variables: i) PC1, referred to as *Participant_Fat_Att*, represents increasing body image concern, together with perceived social pressures about body image from the media, peer groups, and family; and ii) PC2, referred to as *Participant_Musc_Att*, represents perceived social pressure for and positive attitudinal responses towards increasing muscularity, combined with a drive to take part in activities that would achieve this outcome.
- Computation of three linear mixed effects models:
  - MODEL 1: Participants’ PSE responses predicted from participants’ actual BMI, apparent stimulus muscle mass, apparent stimulus muscle tone, participants’ age, Participant_Fat_Att, and Participant_Musc_Att as explanatory variables. See **Figure 3**, upper and middle rows, for illustrated model outcome.
  - MODEL 2: Participants’ PSE responses predicted from participants’ percentage body fat, participants’ muscle mass, apparent stimulus muscle mass, apparent stimulus muscle tone, participants’ age, Participant_Fat_Att, and Participant_Musc_Att as explanatory variables. See **Figure 4** for illustrated model outcome.
  - MODEL 3: Predicted participants’ DL responses using participants’ actual BMI, apparent stimulus muscle mass, apparent stimulus muscle tone, participants’ age, Participant_Fat_Att, and Participant_Musc_Att as explanatory variables. See **Figure 3**, bottom row, for illustrated model outcome.
- Simulation to illustrate how large the differences in body-size estimates (i.e., PSE in BMI_hse_ units) can be in individual participants who have the same actual BMI. To do this, we estimated the covariance between body fat and skeletal muscle mass in men, from a modest database of 178 Caucasian males whose body composition had been measured using a Tanita MC780MA multi-frequency segmental body composition analyzer. See **Figure 5** for illustrated model outcome.
- Simulation to illustrate the likely effect sizes of stimulus muscle mass and muscle tone that we would obtain, *if* we had stimuli that were correctly calibrated for body composition. To do this, we again used the modestly sized body composition database of 178 Caucasian men. This simulation converged on a qualitatively similar pattern of results, even though the sizes of the effects were reduced by ∼40% for stimulus muscle mass and ∼18% for stimulus muscle tone.

## Results

### Univariate Statistics

**Table 1** shows the characteristics of our 45 male participants. With respect to the World Health Organization’s BMI classification scheme (23), the numbers of participants who fell into the under-weight, normal, over-weight, and obese categories were 2, 22, 15, and 6. Performance in the psychometric tasks also fell within the normal ranges for the BPSS-M (36), the SATAQ-4 (37), and the DMS (38). All raw DL scores departed markedly from a normal distribution for each condition (smallest Shapiro-Wilk’s *W* = 0.223, p < .0001) and were therefore logarithmically transformed for further analysis.

Reliability for the yes-no task was computed by taking participant PSE scores from block 2 and block 3 of the psychophysical task output and comparing these for each task level using paired samples *t*-tests. All reliability tests were conducted retrospectively, completed following all data collection. As can be seen in **Table 2**, all paired samples *t*-tests showed no sginificant differences between blocks and all correlations between the paired means were shown to be statistically significant, demonstrating good task reliability for our final sample.

**Table 2:** Results of a paired samples t-tests between the mean PSE scores of block two and block three of the psychophysical task for each task level (N = 45). * = *p* < .001.

Task Level	Correlation	Mean Difference (*SD*)	*T* – value
Low Tone-Low Mass	*.901	–0.44 (2.41)	–1.227
Low Tone-Mid Mass	*.802	–1.08 (3.77)	–1.921
Low Tone-High Mass	*.830	–0.97 (3.25)	–2.004
Mid Tone-Low Mass	*.918	–0.09 (2.26)	–0.282
Mid Tone-Mid Mass	*.806	–0.59 (3.35)	–1.187
Mid Tone-High Mass	*.839	–0.43 (2.97)	–0.971
High Tone-Low Mass	*.794	0.46 (3.24)	0.948
High Tone-Mid Mass	*.787	–0.35 (3.43)	–0.686
High Tone-High Mass	*.871	–0.49 (3.16)	–1.047

### Multivariate Statistics

We wanted to quantify the relationships between participants’ body size estimates (indexed by BMI_hse_), their actual BMI, their body composition, and the stimulus properties. To do this, we used PROC MIXED (SAS v9.4) to build three linear mixed effects models. Two of these models had participants’ PSE as the outcome variable, and one had Log_10_ DL as the outcome. The first model for PSE and the model for Log_10_ DL used participant’s actual BMI, apparent stimulus muscle mass (i.e., low, mid, and high) and apparent stimulus muscle tone (i.e., low, mid, and high) as fixed effects. In the second model for PSE, we replaced participants’ actual BMI with their percentage body fat and muscle mass as fixed effects. In all three models, we wanted to control for any influence of chronological age and the psychometric variables (BPSS-M, SATAQ, and DMS).

In order to avoid the possibility of introducing substantial variance inflation, we first checked for evidence of co-linearity among the psychometric variables. **Table 3** shows the Pearson correlations for these tasks across the sample of 45 participants. **Table 3** shows several substantial and statistically significant correlations between BPSS-M and subtests of the SATAQ and DMS. Therefore, we used PROC FACTOR in SAS v9.4 (SAS Institute, North Carolina, US) to carry out a principal component analysis with rotation, in order to identify any significant latent variable(s) in the psychometric data. We used the factor scores from these component(s) in our statistical models. The Kaiser-Meyer-Olkin (KMO) measure of sampling adequacy (which indicates the degree of diffusion in the pattern of correlations) was 0.80 suggesting an acceptable sample. Two principal components (PC) had Eigen values greater than Kaiser’s criterion of 1 (i.e., 3.35 & 2.24) which, together, explained 70% of the variance. The scree plot showed an inflexion, i.e., Cattel’s criterion, which also justified retaining just two PCs. The residuals were all small, and the overall root mean square off-diagonal residual was 0.07, indicating that the factor structure explained most of the correlations. The factor loadings on BPSS-M and each of the subtests of the SATAQ and DMS for the two PCs are shown in the last two columns of **Table 3**.

**Table 3:** Pearson correlations for the psychometric tasks. The last two columns show the factor loadings on BPSS-M and each of the subtests of the SATAQ and DMS for the two PCs from the principal components analysis.

	STQ Med	STQ Peer	BPSS-M	STQ Fat	STQ Fam	DMS Beh	STQ Musc	DMS Att	PC1	PC2
STQ Med	–								***0.83***	0.15
STQ Peer	0.64***	–							***0.81***	0.25
BPSS-M	0.52***	0.50***	–						***0.77***	–0.05
STQ Fat	0.59***	0.58***	0.44**	–					***0.72***	0.4
STQ Fam	0.37**	0.34*	0.40**	0.18	–				***0.62***	***–0.54***
DMS Beh	0.15	0.23	-0.01	0.27	-0.34*	–			0.04	***0.89***
STQ Musc	0.25	0.35*	0.06	0.42**	-0.25	0.74***	–		0.19	***0.86***
DMS Att	0.26	0.35*	0.20	0.30*	-0.29	0.64***	0.60***	–	0.20	***0.80***
DMS Totl	0.23	0.32*	0.11	0.31*	-0.33*	0.91***	0.74***	0.90***	–	–

* = p < .05, ** = p < .01, *** = p < .001.

NB: STQ Fat, STQ Body Fat; STQ Musc, STQ Muscular; STQ Fam, STQ Family pressure; STQ Peer, STQ Peer pressure; STQ Med, STQ Media pressure; DMS Att, DMS Attitudes; DMS Beh, DMS Behaviors; DMS Totl, DMS Total.

PC1 loaded primarily on to BPSS-M and all SATAQ subtests excluding that for muscularity. We interpreted increasing scores on this PC (henceforth referred to as Participant_Fat_Att) as representing increasing body image concern, together with perceived social pressures about body image from the media, peer groups, and family. PC2 loaded primarily on to the muscularity dimension of the SATAQ, as well as both DMS scales. We therefore interpreted increasing scores on this PC (henceforth referred to as Participant_Musc_Att) as representing perceived social pressure for and positive attitudinal responses towards increasing muscularity, combined with a drive to take part in activities that would achieve this outcome.

Each of the three linear mixed effects models was optimized by ensuring that a) any fixed effect added to a model contributed a reduction in -2 Log Likelihood, b) fixed effects were retained in a model only if their Type III test of fixed effects was significant at p < .05. The only exceptions to this were where one non-significant fixed effect comprised part of a significant two- or three-way interaction term, in which case it was retained. In addition, we permitted individual variation at the intercept level for each participant, by including a random effect for participant. Note, as both stimulus muscle mass and muscle tone comprised three levels (high, mid, and low), we used the high level as the control when dummy coding these variables in each model. The detailed outcome of the statistical modelling is shown in **Table 4** and is illustrated graphically in **Figures 3**, **4**.

**Table 4:** Output from the 3 linear mixed effects models.

Model Parameters	F-value (DF)	Z-value	p-value	Parameter estimate	Parameter 95% CI	-2Log likelihood
**1) Model 1 for PSE**						
Empty Model Full Model Fixed Effects: Stim_Musc_Tone Stim_Musc_Mass Participant_BMI Participant_Musc_Att Participant_Age	6.97 (2, 350) 15.92 (2, 349) 64.35 (1, 44) 9.86 (1, 44) 5.40 (1, 44)		<.001 <.001 <.001 .003 .03	1) 0.50 2) 1.28 1) 0.18 2) 1.76 0.67 –1.18 0.095	–0.18 – 1.17 0.60 – 1.96 –0.50 – 0.85 1.09 – 2.44 0.50 – 0.84 –1.95 – –0.42 0.013 – 0.18	2142.2 2001.4
Random Effect:						
Subject variance		3.97	<.001	4.78		
**2) Model for Log** **_10_** ** DL**						
Empty Model Full Model Fixed Effects: Stim_Musc_Tone Stim_Musc_Mass Participant_BMI Participant_BMI × Stim_Musc_Mass	1.61 (2, 356) 12.74 (2, 356) 3.37 (1, 44.9) 10.34 (2, 356)		0.20 <.001 .07 <.001	1) –0.039 2) 0.015 1) 0.58 2) 0.88 0.034 1) –0.021 2) –0.011	–0.044 – 0.017 –0.046 – 0.076 0.24 – 0.93 0.53 – 1.23 0.014 – 0.054 –0.034 – 0.0071 –0.044 – 0.017	177.4 141.1
Random Effect:						
Subject variance		4.29	<.001	0.068		
**3) Model 2 for PSE**						
Empty Model Full Model Fixed Effects: Stim_Musc_Tone Stim_Musc_Mass Participant_Body_Fat Participant_Musc_Mass Participant_Age	7.40 (2, 309) 11.83 (2, 309) 6.56 (1, 39) 5.80 (1, 39.2) 9.80 (1,39.1)		<.001 <.001 .01 .03 .003	1) 0.53 2) 1.43 1) –0.03 2) 1.56 0.38 –0.21 0.16	–0.20 – 1.26 0.70 – 2.17 –0.77 – 0.70 0.82 – 2.29 0.08 – 0.68 –0.41 – –0.013 0.06 – 0.26	2142.2 1799.9
Random Effect:						
Subject variance		3.91	<.001	7.02		

NB Stim_Musc_Tone, stimulus muscle tone; Stim_Musc_Mass, stimulus muscle mass; Participant_Musc_Att, psychometric latent variable for participants’ attitudes to muscularity.

The top row of **Figure 3** shows scatterplots from the first model for PSE, the index of participants’ body size estimation (in BMI_hse_ units), and corresponds to “Model 1 for PSE” as shown in **Table 4**. Stimulus muscle tone increases across the three plots from the first (left) to the third (right) column. In each graph, values of PSE predicted from the model are plotted on the *y*-axis as a function of participant’s actual BMI. Within each plot, data points and their respective regression lines for PSE on actual BMI are shown for low (black), mid (gray), and high (white) stimulus muscle mass. The black dashed line represents veridical responses, i.e., where a participants’ body size estimate in BMI_hse_ units would exactly match their actual body size in BMI units. Not surprisingly, participants' estimates of their own body size systematically increased with their actual BMI, as many authors have shown before (e.g., 27, 47). A second point to note is that the slopes of the regression lines are all less than 1 (F1,43 = 5.34, p = .03). This is consistent with the yes-no task producing a contraction bias effect (48), as reported previously by 27, 28. This is a perfectly normal bias seen in unanchored magnitude estimation tasks, such as our yes-no task.

The important results for the current study are the significant effects that both the apparent muscle mass and muscle tone of the stimuli have on participants’ body size estimates. Specifically, stimuli that are judged subjectively to have low muscle mass give rise to significantly higher body size estimates than do those judged to have mid muscle mass (LSmean difference = 1.58 BMI_hse_ units, t = 4.61, p < .001) or high muscle mass (Lsmean difference = 1.76 BMI_hse_ units, t = 5.13, p < .001). The difference between body size estimates for mid and high muscle mass stimuli was not statistically significant (LSmean difference = 0.18 BMI_hse_ units, t = 0.52, p = .6). Similarly, stimuli that are judged subjectively to have lower muscle tone give rise to higher body size estimates. The corresponding differences in the LSmeans for body size estimates were: low to mid tone = 0.78 BMI_hse_ units, t = 2.27, p = .02; low to high tone = 1.28 BMI_hse_ units, t = 3.71, p < .001; mid tone to high tone = 0.50 BMI_hse_ units, t = 1.45, p = .1.

The middle row of **Figure 3** illustrates the statistically significant and independent influence that Participant_Musc_Att had on body size estimates. This also is derived from “Model 1 for PSE” as shown in **Table 4**. The graphs in this row follow the same regime as above except that the regression lines for the three different stimulus muscle mass levels are plotted at +1 *SD* (solid lines) and -1 *SD* (dashed lines) for Participant_Musc_Att. These graphs show very clearly that increasingly positive attitudes and drive towards muscularity are associated with higher body size estimates.

The bottom row of **Figure 3** shows scatter-plots of the output from the “Model for Log_10_ DL” in **Table 4**, which indexes the smallest difference in body size that participants can detect; i.e., their sensitivity in the yes-no task. As before, stimulus muscle tone increases across the three plots from left to right and the color coding for stimulus muscle mass is the same. In each graph, predicted values of Log_10_ DL are plotted on the *y*-axis as a function of participant actual BMI. It is clear from all three graphs that sensitivity reduces (i.e., DL increases) with increasing actual BMI. This effect is systematically greater—i.e., the regression slopes are steeper—for stimuli judged to have greater muscle mass. As **Table 4** shows, this effect is statistically significant. There is, however, no significant influence of stimulus muscle tone on DL (**Table 4**). The Weber fractions (i.e., ΔI/I) reduce over the range of participant actual BMI from 15 to 42.5 for low stimulus muscle mass (0.074–0.033), remain approximately constant for mid stimulus muscle mass (0.058–0.047), and increase for high stimulus muscle mass (0.030–0.081). Therefore, participants gave responses which best approximated Weber’s law when viewing stimuli with mid-level muscle mass.

**Figure 4** shows two contour plots derived from the second model for PSE (see **Table 4**, “Model 2 for PSE”), in which the fixed effect of actual BMI was replaced with two fixed effects together constituting body composition: percentage skeletal muscle mass and percentage body fat of the participant. As **Table 4** shows, both of these factors had statistically significant effects on participants’ body size estimates, although these effects were in opposite directions: body size estimates increased with increasing participant body fat and decreased with increasing participant muscle mass. Each plot in **Figure 4** shows predicted PSE in the *z*-axis: grey levels from black to white represent low to high predicted PSE. Participants’ body fat and muscle mass are plotted on the *x*- and *y*-axes respectively. The plot on the left of **Figure 4** corresponds to high stimulus muscle mass and tone. The plot on the right of **Figure 4** corresponds to low stimulus muscle mass and tone. The white dashed line in each plot corresponds to a predicted PSE of 24 BMI_hse_ units. The important point illustrated by **Figure 4** is that variable combinations of participant muscle mass and body fat (i.e., body composition) can give rise to identical estimates of body size, when measured in BMI_hse_ units. However, in order to achieve the same PSE with stimuli of lower muscle mass and tone, this regime shifts to the left.

### How Big Are the Differences in Estimated Bmi_hse_ for Participants Who Have the Same Actual BMI?

The implication from **Figure 4** is that individuals who have the *same* actual BMI, but who have *different* body compositions, will estimate their body size, when indexed in BMI_hse_ units, very differently. This is illustrated by two participants from our dataset, *A* and *B*, in the left pane of **Figure 4**, both of whom have a BMI ∼23. Clearly, in the context of a body size estimation task where only the adiposity of stimuli is changed, this is potentially very undesirable. Therefore, we wanted to quantify just how large this variation in body size estimation can be. In principle, we could achieve this directly if we knew how much variation there is in the body composition of the participants at different actual BMIs. Unfortunately, in our experimental dataset, there were not enough participants whose actual BMI fell within the range of a BMI unit +/- 0.5 to estimate such covariance reliably. Instead, we used a body composition database which was obtained from 178 Caucasian males (age *M* = 33.6, *SD* = 11.15; actual BMI *M* = 25.4 *SD* = 3.75; body fat *M* = 14.4 *kg*, *SD* = 7.29 *kg*; skeletal muscle mass *M* = 39.1 *kg*, *SD* = 5.7 *kg*) using a Tanita MC780MA multi-frequency segmental body composition analyzer. We used this dataset to calculate the covariance between body fat and skeletal muscle mass, at each actual BMI point (+/-0.5 BMI units) for which there were at least 15 observations—i.e., where the covariance estimate is more likely to be reliable. According to this criterion, the covariance values at BMIs 22, 23, 25, and 26 were -0.55, -0.41, -0.37, and -0.17 respectively. Moreover, we had 12 BMI points between BMIs 18–31 for which we had at least 5 data points, and the average covariance across these 12 points was *M* = -0.35, *SD* = 0.23. We then used PROC SIMNORM in SAS v9.4 (SAS Institute, North Carolina, US) to calculate 5 bivariate normal distributions, each with 10,000 data points, for a range of covariance values from -0.15 to -0.55 in steps of 0.1, consistent with the covariance values that we observed in the data at different BMIs. Next, for each of these 5 distributions, we computed prediction ellipses that captured 50%, 60%, 70%, 80%, and 90% of the possible combinations of percentage body fat and skeletal muscle mass—i.e., from about half of the range in each distribution to almost the full range, as is illustrated in **Figure 5A**. In the final step, separately for each of the five distributions, we identified from these ellipses the biggest difference in body composition at each of the prediction values (i.e., lowest body fat with highest muscle mass and vice versa) and used Model 2 for PSE (see **Table 4**) to convert these participant body composition values into self-estimates of body size, expressed in BMI_hse_. **Figure 5B** shows plots of the difference in these pairs of BMI_hse_ estimates (*y*-axis), as a function of prediction ellipse percentage (*x*-axis). Separate lines are plotted for the five different covariances between body fat and skeletal muscle mass. The white band in the background highlights the most plausible range of BMI_hse_ differences, given that it selects prediction ellipses that capture most but not all combinations of body fat and muscle mass computed from covariance values in the middle of the range that we observed in real data. What is striking is that even a conservative evaluation of these simulations forces the conclusion that differences in body size estimation by participants who have the same actual BMI are large, typically between ∼5–7 BMI_hse_ units, which is enough to leapfrog between body weight classifications in World Health Organisation, (23) criteria.

### Applications of Real Skeletal Muscle Mass Values to Correct Bmi_hse_ Estimates of Body Size

As a final step in our analyses, we attempted to assign plausible muscle mass values to our stimuli (as distinct from qualitative labels) and recalculate the effects of stimulus muscle mass and tone on body size estimates. If our strategy for calibrating stimuli for BMI_hse_ is completely unrelated to reality, then we should expect to see our error estimates all but disappear. If however the analyses we present have some validity, we should expect to see similar effects once plausible muscle mass values have been assigned.

As described in the *Methods* section, we generated low, mid, and high muscle content bodies by setting the morph dimensions of muscularity and muscle tone in Daz Studio to either low, mid, or high levels. Therefore, to assign plausible low, mid, and high muscle mass values in kg to each stimulus class, we first divided the distribution of skeletal muscle mass values from our biometric database of 178 men into three ranges split at the 33rd and 67th centiles (low mid and high skeletal mass means were: *M* = 33.67 kg, *SD* = 2.48; *M* = 39.00 kg, *SD* = 3.30; *M* = 44.81 kg, *SD* = 4.20, respectively), and assigned a categorical variable with three levels to correspond to these three ranges. We then used PROC MIXED in SAS v9.4 to predict actual BMI in this database from i) the centile to which a skeletal muscle mass belonged, ii) an individuals’ waist circumference, and iii) an individual’s hip circumference. The fitted model thus allowed us to connect the biometric database to our experimental dataset because, for every body-size estimate in BMI_hse_ units, we know the waist and hip circumference of the corresponding CGI model. For example, for a high muscle mass, mid muscle tone stimulus, we can enter the waist and hip values that correspond to a body size estimate in BMI_hse_ units into the fitted model from the biometric database and calculate what the body size estimate would be in real BMI units. As a final step, having converted every body-size estimate from BMI_hse_ to real BMI units in this way, we re-ran model 1 in **Table 4**. We found significant Type III fixed effects for: stimulus muscle mass (F2,349 = 8.11, p < .001), stimulus muscle tone (F2, 350 = 6.63, p = .001), participant age (F1,44 = 5.41, p = .02), participant actual BMI (F1,44 = 64.36, p < .001), and Participant_Musc_Att (F1,44 = 9.86, p = .003).

*Post hoc* pairwise comparisons still showed that low muscle mass stimuli gave rise to significantly higher body size estimates than did mid muscle mass (LSmean difference = 0.89 corrected BMI_hse_ units, t = 3.08, p = .002) or high muscle mass stimuli (LSmean difference = 1.09 corrected BMI_hse_ units, t = 3.79, p < .001). The difference between body size estimates for mid and high muscle mass stimuli was not statistically significant (LSmean difference = 0.21 corrected BMI_hse_ units, t = 0.71, p = .5). With respect to muscle tone, the corresponding differences in the LSmeans for body size estimates were: low to mid tone = 0.63 corrected BMI_hse_ units, t = 3.08, p = .03; low to high tone = 1.05 corrected BMI_hse_ units, t = 3.62, p < .001; mid tone to high tone = 0.42 corrected BMI_hse_ units, t = 1.47, p = .1. In short, assigning plausible muscle mass values to our stimuli gave rise to a qualitatively similar pattern of results, even though the sizes of the effects were reduced by ∼40% for stimulus muscle mass and ∼18% for stimulus muscle tone.

## Discussion

The primary aim of this study was to estimate how much variation there is in men’s own body size estimates, when measured in BMI_hse_ units, caused by i) variation in the participants’ own body composition and ii) variation in the apparent muscle mass and muscle tone of the stimuli being judged. Our results suggest that the accuracy of male body judgments is not captured using body stimuli which only vary in adiposity, but instead needs variation in both adiposity and muscularity to accurately represent the perception of body image and reflect the variation of these dimensions in the male population.

Consistent with previous studies where women estimated their own body size or other women’s body size [e.g., Refs. (27, 47, 49)], in the current study, plots of estimated body size are linearly predicted by the participant’s own actual BMI, but with a slope of less than unity (see the top two rows of **Figure 3**). Lower actual BMI participants over-estimate body size, middle-range actual BMI participants’ estimates are the most accurate, and high actual BMI participants under-estimate. This pattern of responses is predicted by a normal perceptual feature of magnitude estimation called contraction bias (48). It occurs when the psychophysical task is not anchored, which means that the participant does not have available to them constant reminders of the smallest and largest examples from the range of stimuli they will be presented. In this situation, body size estimation must be made by comparing the difference between the size of the stimulus presented to the body size the participant believes themselves to have with an internal reference distribution based on all the bodies that the participant has ever seen. This kind of judgment is most accurate when the participant’s belief is closest to the average body size of their internal reference distribution, and increasingly less accurate as the two diverge. When there is an increasing difference between the reference and the body size being estimated, the participant makes an estimate closer to the average of the reference distribution than it should be. Hence, the term contraction bias (48).

In addition, the ability to detect a change in body size (as indexed by the DL) becomes progressively worse as the BMI_hse_ of the bodies being judged increased (see the bottom row of graphs in **Figure 3**). This is consistent with another feature of perception called Weber’s law. Weber’s law states that the just noticeable difference (JND) between two stimuli will be a constant proportion of their magnitude, leading to a constant Weber fraction over the stimulus range (46). This means that discriminating between higher BMI_hse_ bodies requires progressively larger differences in BMI_hse_ between stimuli (29).

### Psychological Attitudes

As in previous studies with female participants, the psychological state of the participants modulates the accuracy of their self-estimates of body size [e.g., Refs. (27, 47]. In the current study, this is an effect that was statistically independent of their perceptual responses and is consistent with a multidimensional model of body image in which the size and shape someone believes themselves to be is a linear combination of attitudinal and perceptual factors [cf. (1)]. We found that men who have increasingly positive attitudes and drive towards muscularity were more likely to over-estimate their body size. However, by contrast to previous findings with female participants, body fat concerns did not influence the male participant’s judgments. This may reflect a difference in the relative importance of muscularity and body fat in men and women. Body fat has been consistently identified as the central feature of body image concerns in women, whereas in men the central concern has been identified as muscularity [e.g., Refs. (19, 50–54). This is reinforced by a strong social media pressure to be both high in muscularity and low in adiposity (55, 56). Additionally, concerns about muscularity, along with concerns with adiposity, are suggested to play a key role in the development of anorexia nervosa in men (14), emphasizing the need to be able to independently index body image concerns about muscularity and adiposity to determine their separate importance in its etiology.

### Apparent Muscle Mass and Muscle Tone of the Stimuli

Looking *across* the stimulus types, our results suggest that as apparent muscle mass and muscle tone *decrease* in the stimuli, so men effectively selected images with higher BMI_hse_ values to match the body size they believe themselves to have. This is an important result in several ways. First, it gives some insight into how the men may have been solving the task. Our stimulus calibration procedure is based on a multiple regression equation derived from anthropometric measurements obtained from the Health Survey for England, specifically waist and hip circumferences. This means that in our set of CGI bodies, a stimulus that has a BMI_hse_ of 25 will have exactly the same waist and hip circumference irrespective of which combination of low/mid/high muscle mass and low/mid/high muscle tone it comprises. Therefore, according to our first hypothesis, if our participants had been using the horizontal widths across the waist-hip region to match their own body size belief against the stimulus [cf. (31)], then we would not have found statistically significant differences in body size estimates between the different levels of stimulus muscle mass and tone. Given that men are more likely to deposit fat on the stomach than women (57, 58), fixating this region for estimating adiposity would be an even better strategy for men than for women. This is because the men would have reliably selected the same matches across muscle mass/tone combinations for a given belief about their own body size (i.e., they would have chosen the bodies with the same waist and hip widths). Had this been the case, graphically we would have seen the black, white, and gray regression lines in the first two rows of **Figure 3** overlie each other. But they do not. Instead the self-estimates of body size were ∼2.5 BMI_hse_ units greater for the low muscle mass stimuli than either the mid or high muscle mass stimuli, and this is consistent with our second hypothesis: that men may attend to the chest and upper arms when matching stimuli to the body size/shape they believe themselves to have.

Critically, when we recalculated these effects, having attempted to assign plausible skeletal muscle mass to our stimuli, we observed the same pattern of results, albeit the effect sizes were reduced by up to ∼40%. This provides convergent evidence that reinforces the need for all researchers to be running these kinds of experiments with stimuli that are correctly calibrated for body composition and BMI.

From a practical point of view, constructing a figural scale for body-size estimation where *only* adiposity changes would mean that an arbitrary choice would need to be made about the apparent muscularity of the stimuli presented to participants. The present results show that an arbitrary choice of this kind could lead to fixed errors in any survey results using such a scale. For example, suppose two figural scales were developed, one from our low muscle mass/low muscle tone images and the second from our mid muscle mass/mid muscle tone images. We would expect to see, on average, that self-estimates of BMI_hse_ would be ∼2.5 BMI_hse_ units higher for the former scale, and this could lead in turn to over-estimates of obesity rates, for example. Similarly, research highlights that there is a comparable split between males who wish to lose weight, and those seeking to gain weight (16–18). Presenting a stimulus set with an arbitrary choice of visual muscularity would introduce considerable uncontrolled variability into any epidemiological study or public health assessment. In a clinical sample, e.g., men with eating disorders or muscle dysmorphia, such erratic body size estimation may even compromise the effective intervention and treatment of body image distortion (54).

### Participant Body Composition

We calculated the potential variation in self-estimates of body size, when measured in BMI_hse_ units, that is attributable to the body composition of the participant. To facilitate these calculations, we needed sensible estimates of the covariance between body fat and skeletal muscle mass as a function of actual BMI. We obtained these covariance estimates from a bio-impedance database of 178 male volunteers and used them in a simulation to identify a range of maximum differences in body composition in individuals who would have the same BMIs. As a last step, we entered these body composition values into our fitted model from the experiment which predicts body size estimates in BMI_hse_ units from the body composition of the participant, and calculated the predicted differences in body size estimates. The results are illustrated in **Figure 5B**. For participants with the *same* actual BMI, the results show that self-estimates of body size can potentially vary over a range of ∼5–7 BMI_hse_ units based on differences in the skeletal muscle and fat composition of the participant. This suggests a strong potential source of uncontrolled variance in body size estimation when using body scales which are designed to vary only in adiposity. Errors of this magnitude can easily move a participant’s self-estimate of BMI_hse_ between BMI categories, such as from normal to overweight or even to obese.

This study strongly suggests that for men’s bodies, stimuli that do not account explicitly for variation in both muscle mass and muscle tone in the stimuli, as well as measurement methods that do not take explicit account of body composition in the participant, may lead to significant errors in self-estimates of body size. This leads to the important question of whether a similar problem exists for self-estimates of body size in women. Although women’s bodies tend to show a lower degree of variation in their proportion of muscle to fat than male bodies, the increase in resistance training in fitness and exercise regimes has increased this variation. Moreover, the trend towards “fitspiration,” a lean and toned body rather than just a low-fat body, has created a strong media and social pressure to achieve an athletic ideal [e.g., Refs. (59, 60)]. In addition, in women with an eating disorder such as anorexia nervosa, there is significant variation in body composition linked to the severity of their condition (61, 62). Currently for women, test stimuli usually vary only in simulated adiposity, but our results suggest that this may not be a very accurate way of assessing women’s perception of body size. Future studies should determine whether self-estimates of body size are affected by the female participant’s body composition and whether their perception of their body size can be more accurately captured by varying the body stimuli used to index their judgment in multiple dimensions, such as adiposity and muscularity.

### Is There a Solution?

In this study, healthy men made nine self-estimates of body size, expressed in units of BMI_hse_. To do this, they used the same yes-no task nine times, but on each task run, all the stimuli for that run represented a different combination of apparent muscle mass and tone. During each task run, only the adiposity of the men in the stimuli varied. Therefore, the participants were essentially picking what level of adiposity in the stimulus, for a fixed combination of muscle mass and tone, matched the body size they believed themselves to have. We found that variation in both the apparent muscle mass and tone in the stimuli, as well as individual variation in the body composition of the participant, led to far reaching differences in body size estimation, when expressed in BMI_hse_ units. Qualitatively, we replicated these effects when we assigned plausible real muscle mass values to our stimuli. This not only confirms but also quantifies to some extent what researchers in this field have long suspected: that BMI has limited utility as a metric for body size estimation in men. So, the question is, if not BMI, then what? One obvious alternative is to have a stimulus set that represents variation in both muscle mass and adiposity parametrically. This way, participants can match the body *shape* they believe themselves to have to the stimulus on offer. Clearly, some authors have already gone down this route with the use of line-drawn images based on the somatomorphic matrix (25, 63) as well as CGI versions of the same (26). However, it is not clear that there is an accurate and calibrated mapping in these stimuli between the body shapes illustrated and the adiposity and muscle mass they are supposed to represent. One way to improve on this situation, therefore, would be to combine body composition measurements from bio-impedance or dual-energy X-ray absorptiometry (DXA) with 3D body shape scanning techniques in a large sample of volunteers. Such a dataset could be used to reveal the statistical mapping between 3D body shape change as a function of muscle mass and adiposity, and these statistical models could be used in turn to create appropriately calibrated 3D CGI models of men. Such stimuli could then be incorporated into a method of adjustment task in which both dimensions of muscle mass and adiposity could be manipulated simultaneously by mouse control. Development of such stimulus sets are vital in providing comparable measurements of the male body within size estimation tasks. Not only will the achievement of this allow for much needed progress in understanding the etiology of body image distortions in men but will provide headway in the development of gender specific interventions for men with body image disorders. Initial steps have been taken along this route in studies of body size estimation in men and women by combining 3D body shape scans with BMI measures (28, 64–66), but clearly need to be extended to allow manipulation of body composition.

In conclusion, this study suggests that the accuracy of male body judgments cannot be captured simply using body stimuli only varying in adiposity, but instead requires variation in both adiposity and muscularity to accurately index the perception of body image and reflect the significant variation in these dimensions in the male population.

## Data Availability Statement

The datasets generated for this study are available on request to the corresponding author.

## Ethics Statement

This study was carried out in accordance with the recommendations of the relevant guidelines and regulations set out by the local ethics committees at Northumbria University and the University of Lincoln with written informed consent from all subjects. All subjects gave written informed consent in accordance with the Declaration of Helsinki. The protocol was approved by the ethics committees at Northumbria University and the University of Lincoln.

## Author Contributions

All authors contributed to the planning, data collection, and write-up of the study.

## Conflict of Interest

The authors declare that the research was conducted in the absence of any commercial or financial relationships that could be construed as a potential conflict of interest.
